# Supplementary material for: Investigation of the Nanoparticulation Method and Cell-Killing Effect following the Mitochondrial Delivery of Hydrophobic Porphyrin-Based Photosensitizers
Source: Int J Mol Sci. 2024 Apr 12;25(8):4294. doi: 10.3390/ijms25084294 (PMC11050504; doi:10.3390/ijms25084294)
Supplement: Supplementary file 1 [file ijms-25-04294-s001.zip › ijms-2915210-supplementary.pdf]

Supporting Information

Materials and methods

Preparation of rTPA@MITO-Porter using iLiNP. The settings for the microfluidic device are presented in Figure 3. As presented in the image, the invasive Lipid Nanoparticle Production device (iLiNP) [11] contains the basic structure of 10 baffle mixer structure sets. The standard dimensions of the baffle mixer structure were a width of 150 μm, a depth of 100 μm, and an interval of 100 μm. An EtOH solution containing 1.3 mM lipids [DOPE:Chol:DSPE-PEG2000:rTPA (9:2:0.22:X, molar ratio)] and phosphate-buffered saline [PBS (−)] were prepared. We prepared rTPA-MITO-Porter with two different Drug/Lipid ratios (Drug/Lipid = 0.5[mol%] for Low-MP, Drug/Lipid = 2.5[mol%] for High-MP). When rTPA@MITO-Porter was labeled with a fluorescent probe, 0.5 mol% NBD-DOPE per total lipid were added to the EtOH solution.

rTPA@MITO-Porter was prepared by mixing the lipids in ethanol and PBS (−) using the microfluidic device system at a combined flow rate of 500 μL/min [30% ethanol concentration: 150 μL/min for the lipid phase and 350 μL/min for PBS (−)]. Syringe pumps (Harvard Apparatus, Holliston, MA, USA) were used to control the flow rate. The resulting suspension was then dialyzed for at least 2 h against PBS (−) using Spectra/Por 4 dialysis membranes (molecular weight cutoff 12,000–14,000 Da; Spectrum Laboratories, Rancho Dominguez, CA, USA). After the dialysis, the lipids were modified with 10 mol% STR-R8. The particle size, polydispersity index (PDI), and ζ-potential of the prepared particles were measured using Zetasizer Nano ZS (Malvern Panalytical, Worcestershire, UK). PDI, a measure of the extent of particle size distribution, ranged from 0 to 1. The encapsulation efficiency was calculated using the equation previously reported [11]

Photoinduced $^1O_2$ generation detection. The detection of $^1O_2$ production using SOSG reagent was performed following the manufacturer's recommended protocol. In brief, a mixture of 1.0 μM rTPA in nanocarriers and 5.0 μM SOSG solutions in PBS (−) was irradiated using a xenon lamp (MAX-303, Asahi Spectra; Tokyo, Japan) at a wavelength of 700 ± 6 nm (12.5 mW/cm^2). The change in fluorescence intensity of the solution at 530 nm was measured using a spectrofluorometer with an excitation wavelength of 490 nm.

Cellular uptake analysis using a fluorescence-activated cell sorter. HeLa cells (2 mL, 1 × 10^5 cells/mL/well) were seeded in six-well plates (Corning) at 24 h before the experiment and incubated in 5% CO2 at 37°C. After the cells were washed with 1 mL of PBS (−), they were incubated with DMEM (FBS−), and rTPA@MITO-Porter labeled with NBD-DOPE containing 27.5 μM total lipid was added for 1 h. After two washes with 500 μL of PBS (−) containing heparin (20 U/mL), the cells were trypsinized and suspended in DMEM (FBS+). After centrifugation (700 × g, 4°C, 3 min), the supernatant was removed, and the pellet was suspended in PBS (−) containing 0.5% bovine serum albumin and 0.1% sodium azide. The cell suspension was filtered through a nylon mesh followed by analysis via flow cytometry (CytoFLEX; Beckman Coulter Inc., Pasadena, MA, USA). NBD-DOPE was excited with 488-nm light, and the 525/40 nm filter of the fluorescence detection channel was set for NBD-DOPE.

Intracellular observation by CLSM. The cells were seeded in 35-mm glass-bottom dishes (IWAKI, Tokyo, Japan) 24 h before the experiment (2 mL, 8 × 10^4 cells/mL, incubated at 37°C in 5% CO_2). After washing with 1 mL of DMEM (FBS−), the cells were incubated with DMEM (FBS−) in the presence of the rTPA@MITO-Porter–modified NBD-DOPE containing 27.5 μM total lipid for 1 h. The medium was replaced with 1 mL of fresh DMEM (FBS+), and the cells were then incubated for

1h 40min. After the incubation, the cells were stained with MitoTracker Deep Red (1 mL, final concentration, 100 nM) for 20 min before observation. The cells were washed with DMEM (FBS+), and 1 mL of fresh DMEM (FBS+) was added, after which CLSM images were obtained. The cells were excited with 473-nm light and 635-nm light from an LD laser. Images were obtained using a Nikon-A1 microscope (NIKON CORPORATION, Tokyo, Japan) equipped with a water-immersion objective lens (UPlanSApo 60/NA. 1.2) and a dichroic mirror (DM405/473/559/635). The two fluorescence detection channels (Ch) were set to the following filters: Ch1, 490/50 (green) for NBD-labeled liposome; and Ch2, 660/10 (red) for MitoTracker Deep Red.

Statistical Analysis. Data are expressed as the mean ± SD for the indicated number of experiments. For multiple comparisons, we performed ANOVA, followed by the SNK.

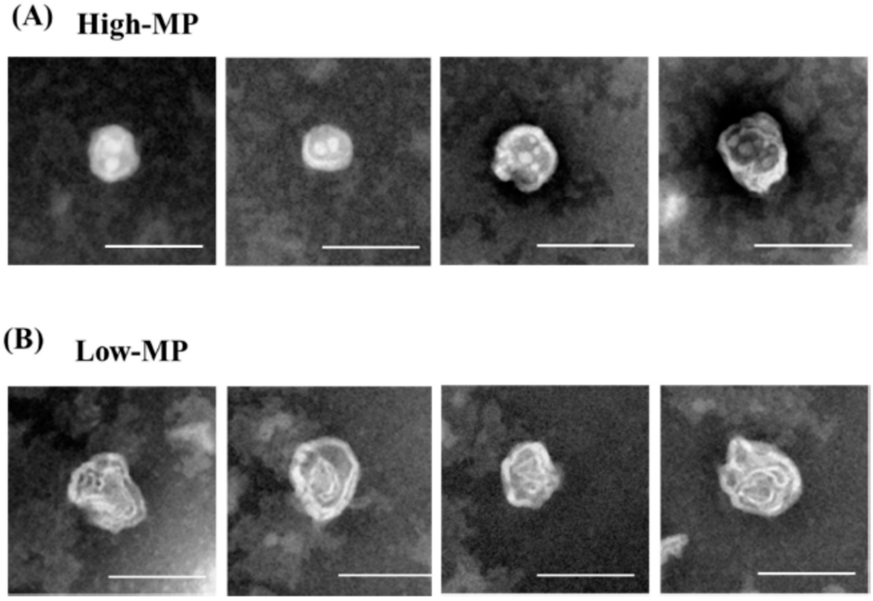

Figure S1. TEM images. (A) High-MP, (B) Low-MP. Scale bar, 100 nm (pictures are from Ref. 6)

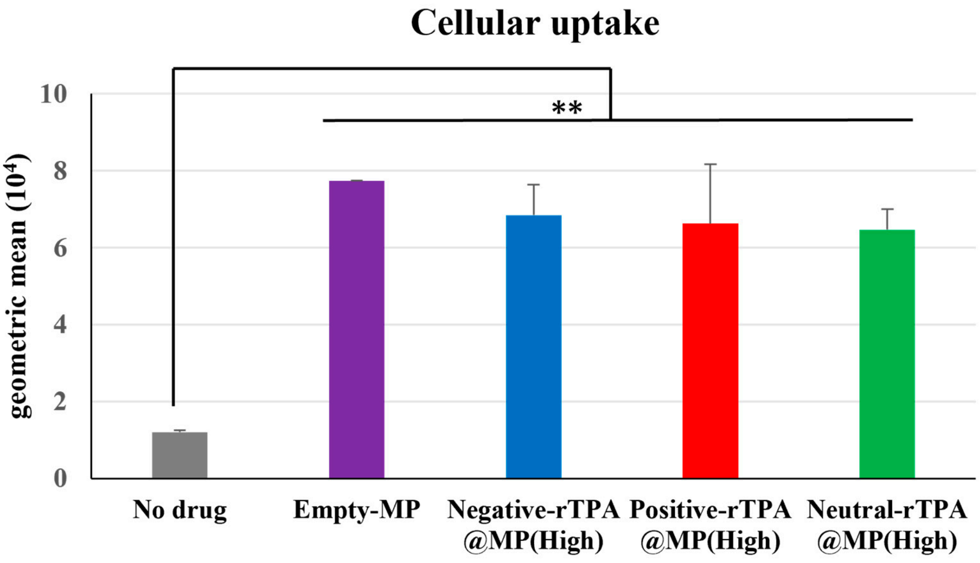

Figure S2. Flow cytometry to evaluate the cellular uptake of High-MP. Cellular uptake is expressed as the mean fluorescence intensity. Data are presented as the mean ± SD (n = 3; **$p < 0.01$ by non-repeated ANOVA, followed by SNK.

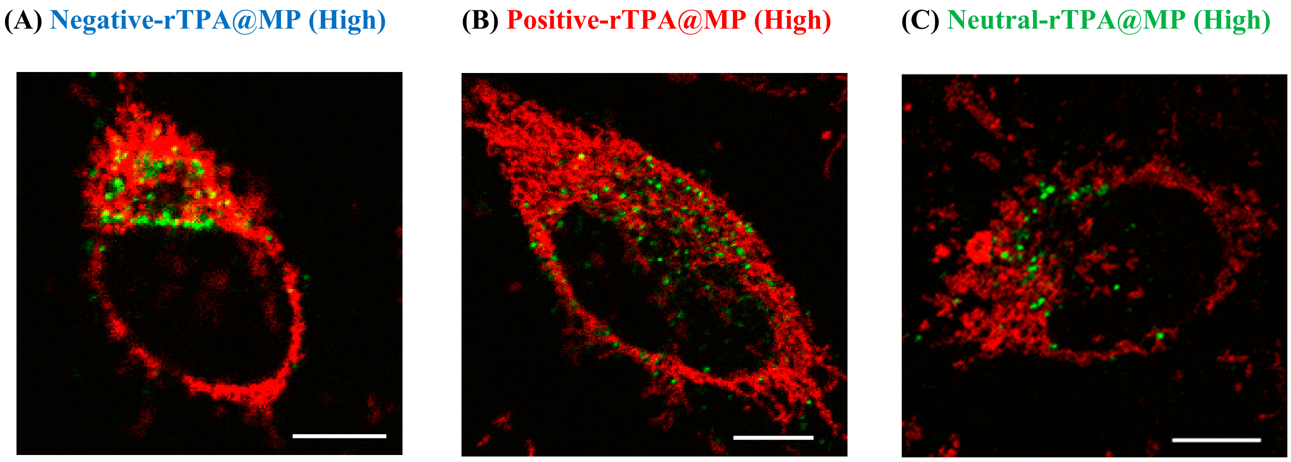

Figure S3. Intracellular observation of High-MP by CLSM. MITO-Porter (green color) co-localized with red-stained mitochondria in HeLa cells, as indicated by yellow signals in the merged image. Scale bar, 10 µm.

(A) rTPA-NH$_2$

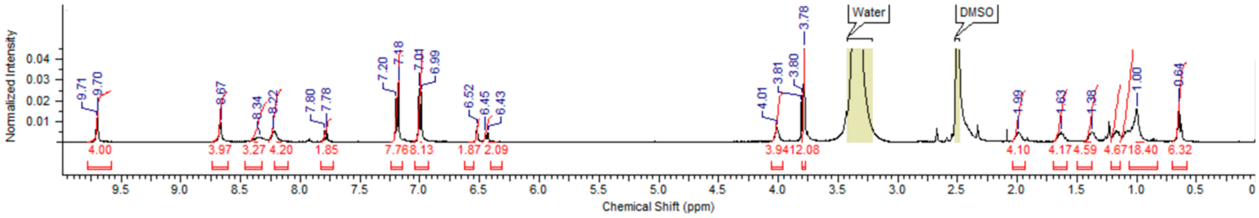

(B) rTPA-OH

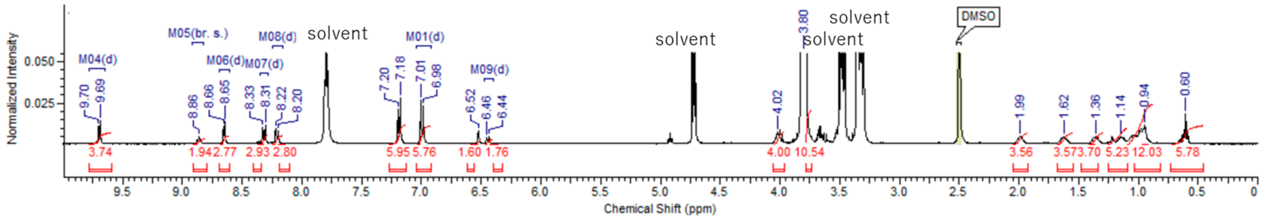

Figure S4. The 1H NMR spectra of rTPA-NH$_2$ (A) and rTPA-OH (B). The spectra do not show prominent peaks due to impurities and the purity of the compounds can be guaranteed to be greater than 90%, even including the common errors that arise from NMR measurements (typically ca. 5 %).

References

6. Karges, J., Heinemann, F., Jakubaszek, M., Maschietto, F., Subecz, C., Dotou, M., Vinck, R., Blacque, O., Tharaud, M., Goud, B., Viñuelas Zahı Nos E, Spingler, B., Ciofini, I., Gasser, G., (2020). Rationally Designed Long-Wavelength Absorbing Ru(II) Polypyridyl Complexes as Photosensitizers for Photodynamic Therapy. J am Chem Soc, 142 (14), 6578-6587. doi: 10.1021/jacs.9b13620.

11. Kubota, F.; Satrialdi; Takano, Y.; Maeki, M.; Tokeshi, M.; Harashima, H.; Yamada, Y. Fine-tuning the encapsulation of a photosensitizer in nanoparticles reveals the relationship between internal structure and phototherapeutic effects. J. Biopho-tonics 2023, 16, e202200119. https://doi.org/10.1002/jbio.202200119.
